# Using human-centered design to develop and implement a pediatric mental health care access program

**DOI:** 10.3389/fpsyt.2023.1283346

**Published:** 2024-01-08

**Authors:** Chuan Mei Lee, Joan Jeung, Juliet C. Yonek, Mahmoud Farghal, Petra Steinbuchel

**Affiliations:** ^1^Child and Adolescent Psychiatry Portal, Department of Psychiatry and Behavioral Sciences, University of California, San Francisco, San Francisco, CA, United States; ^2^Clinical Excellence Research Center, Stanford University School of Medicine, Palo Alto, CA, United States; ^3^Department of Pediatrics, Division of Developmental Medicine, University of California, San Francisco, San Francisco, CA, United States

**Keywords:** human-centered design, child psychiatry, pediatrics, primary care, delivery of health care, integrated, pediatric mental health care access program, child psychiatry access program

## Abstract

In 2019, the University of California at San Francisco (UCSF) launched the Child and Adolescent Psychiatry Portal (CAPP), a pediatric mental health care access (PMHCA) program providing remote mental health consultation services to pediatric primary care providers (PCPs) throughout Northern and Central California. The development and implementation of CAPP was guided by Human-Centered Design (HCD), an iterative, rapid-paced innovation process focusing on stakeholders’ needs and experiences, which shaped the development of CAPP’s programs. The resulting key programmatic elements are designed for pediatric workforce development: (1) PCP consultation with a child and adolescent psychiatrist via a telephone warmline; and (2) training and education for providers. CAPP has grown rapidly since its launch, having enrolled 1,714 providers from 257 practices spread across 36 counties and provided 3,288 consults on 2,703 unique lives as of August 2023. Preliminary evaluation findings indicate high PCP satisfaction with CAPP’s services, despite continued challenges of integrating behavioral health into primary care. Throughout the HCD and implementation process, multidisciplinary partnerships have proven critical in providing end-user input to inform and improve program design. This growing network of partnerships, developed through the cultivation of personal relationships and trust over time, has also proven essential for CAPP’s rapid growth and sustainability. Overall, this Community Case Study highlights the critical role of partnerships and the importance of taking a people-centered approach, as captured in CAPP’s motto, “Connecting for Care.”

## 1 Introduction

An estimated 16.5% of youth under age 18 years have at least one mental health disorder (1). Despite the high prevalence, only half of youth needing mental health treatment receive care from a mental health professional (1, 2). One significant barrier to treatment is the limited availability of specialty mental health care for children and adolescents, resulting in delays in care and inadequate care (3). A shortage of mental health specialists has long been a challenge for healthcare systems nationwide (4–6) and was further exacerbated during the coronavirus disease 2019 (COVID-19) pandemic, when school-based mental health services were shuttered, and youth were socially isolated (7, 8).

More than ever, pediatric primary care providers (PCP) play a critical role in meeting youth mental health needs (9). PCPs often have a longitudinal relationship with patients and therefore are in a unique position to apply chronic care principles (10) to mental health problems. Furthermore, many youth and families prefer seeking mental health care from their PCP (11). However, many pediatric PCPs do not feel comfortable diagnosing or managing mental health conditions; do not feel that mental health services are within their scope of practice; and/or do not feel they have time to address mental health problems in the primary care setting (12, 13). Improving primary care provider capacity to deliver mental health services is one way to increase access to pediatric mental health care. Pediatric mental health care access (PMHCA) programs, also commonly known as child psychiatry access programs (CPAP), have spread across the United States (US) to support pediatric PCPs in managing mental health conditions in primary care settings (14, 15). Most PMHCA programs provide PCPs with telephone consultation services with a child and adolescent psychiatrist or other mental health specialist, care coordination services (e.g., referrals to local mental health resources), and continuing medical education on mental health topics (16). While further research on PMHCA programs is needed, early findings are promising. One study from the Massachusetts Child Psychiatry Access Program (MCPAP), the longest existing program of this type, found that 50% of parents noted improvement in their child’s situation following PCP consultation by MCPAP (25% “strongly agree,” 25% “agree”) (17). Overall, literature reviews of PMHCA programs have reported high growth in program adoption and high provider and caregiver satisfaction with PMHCA services (14–16).

In September 2019, faculty at the University of California, San Francisco (UCSF) launched a new PMHCA program to address gaps in pediatric mental health care access in California—the UCSF Benioff Children’s Hospitals Child and Adolescent Psychiatry Portal (CAPP). It was started with philanthropic seed funding, which then enabled state and federal funding that have supported CAPP’s growth and expansion throughout northern California. Currently, CAPP provides remote, mental health consultation and education services to pediatric PCPs in 48 counties throughout Northern and Central California. The development and implementation of CAPP was guided by Human-Centered Design (HCD), also known as Design Thinking or User-Centered Design principles in a relationally driven process.

This Community Case Study aims to: (1) describe the HCD process for developing and implementing the CAPP program, (2) describe the resulting key programmatic elements designed to close gaps in access to timely, evidence-based mental health care, and (3) highlight the critical role of partnerships that provided end-user input, outreach, and advocacy, enabling successful implementation and rapid growth.

## 2 Methods

### 2.1 Program setting and context

The CAPP program service area encompasses 48 Northern and Central California counties, where approximately 3.5 million of California’s 8.9 million children reside (18). These counties are characterized by tremendous geographic, socioeconomic, and cultural diversity: 40% of the population identifies as Latinx, 35% as Caucasian, 14% as Asian American or Pacific Islander, 5% as Black, 5% as multiracial, and fewer than 1% as Native American or Alaska Natives (19). Counties range from heavily urbanized (i.e., counties within the San Francisco Bay Area) to rural (i.e., throughout California’s Central Valley and north of the Bay Area). Numerous counties and cities within CAPP’s geographic service area are noted to have extreme poverty for young children, including all of Lake and Mendocino counties (20). In 2019, nearly 4 in 10 children in California were insured by Medi-Cal, California’s Medicaid program (21).

California faces a significant shortage of pediatric mental health clinicians. Close to a third of California’s counties have no child and adolescent psychiatrists (22). While the greater Bay Area has slightly higher than state averages of licensed mental health professionals, including child psychiatrists, the Northern and Sierra Regions have 40% fewer psychologists and psychiatrists than the state average, and the San Joaquin Valley has 80% fewer (23). Meanwhile, mental health needs are highly prevalent; between 2016 and 2020, rates of depression and anxiety among California children aged 3–17 years increased 70%, compared to 26% nationwide (24). Suicide rates and adverse childhood experience scores in multiple Northern California counties are among the highest in the state (25, 26). Yet, as many as two-thirds of California’s children with depression do not receive treatment (27).

### 2.2 Human-centered design process

We chose HCD among other approaches (e.g., Health Network, Collective Impact) to guide the development and implementation of the CAPP program because HCD is a relationally-driven innovation process that focuses on stakeholders’ needs and experiences and uses rapid, iterative, problem-solving phases to develop solutions. HCD was initially popularized in the engineering and business fields but more recently has been adapted for use in healthcare to improve access and outcomes (28). For example, HCD methodology has been applied to develop a perinatal care program for Medicaid-insured individuals (29), improve a guideline-based workflow for prescribing antipsychotics to youth (30), and adapt a nurse-led intervention to reduce cardiovascular risk among people living with HIV (31).

**Figure 1:**
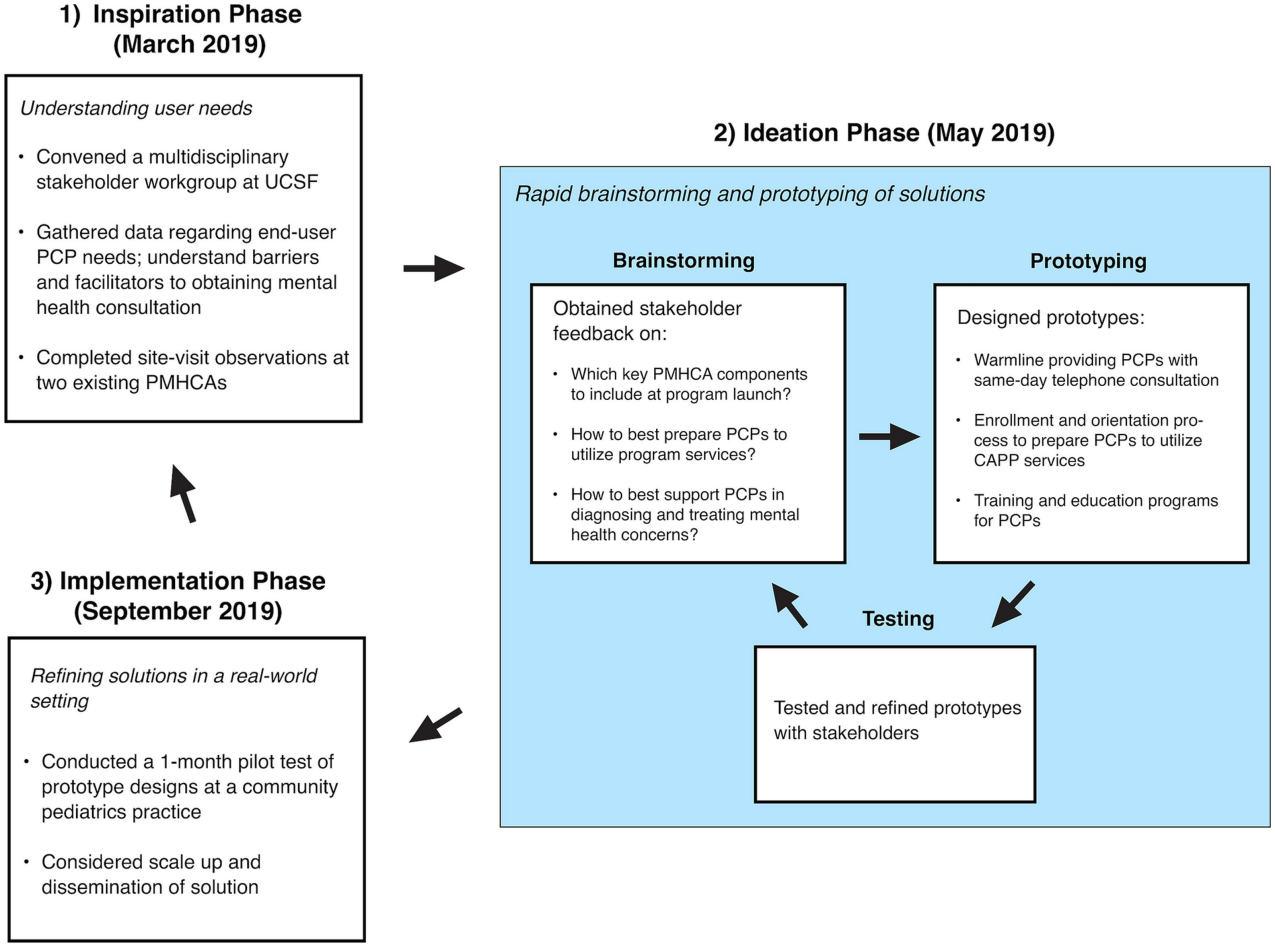
CAPP’s human-centered design process. Figure adapted from Altman et al. (28).

The HCD process consists of three iterative phases: (1) Inspiration, (2) Ideation, and (3) Implementation (Figure 1) (28). First, the Inspiration phase uses qualitative methods to understand the needs and lived experiences of people facing a problem. The second phase, Ideation, involves rapid-paced brainstorming and prototyping of solutions, with a focus on participatory feedback from stakeholders. The third phase, Implementation, continues testing and refining solutions in a real-world setting.

We started the Inspiration phase in March 2019 by convening a Stakeholder Workgroup. This Stakeholder Workgroup was comprised of 11 members, which included 2 CAPP faculty facilitators (CML and PS, who are child and adolescent psychiatrists), 4 pediatric PCPs from federally qualified health centers (FQHC) and academic practices, as well as 5 healthcare system leaders from general and developmental pediatrics, child and adolescent psychiatry, and child psychology. The CAPP faculty facilitators also attended in-person site visits at the Massachusetts Child Psychiatry Access Program (MCPAP) and Washington State’s Partnership Access Line (PAL) to speak with PMHCA leadership and staff members to better understand details of these seminal programs’ structure, processes and operations, as well as key clinical and educational considerations. We then conducted a 90-min focus group with 7 PCPs from one UCSF-affiliated community pediatrics practice serving 70% Medicaid insured patients to gather data on user needs and to understand barriers and facilitators in obtaining mental health consultation. To facilitate participation among busy PCPs, the focus group took place during the practice’s regular weekly meeting time and provided lunch for all participants. Focus group interview questions probed how PCPs were managing mental health concerns within primary care, referral processes to specialty mental health, barriers and facilitators to accessing mental health care for patients, possible barriers and facilitators to utilizing PMHCA consultation services, and PCP needs in mental health continuing education.

From the Inspiration phase of the HCD process, we acquired two core insights: (1) pediatric specialty mental health access remains challenging even in a relatively well-resourced metropolitan area like the San Francisco Bay Area; and (2) pediatric PCPs welcomed continued workforce development to improve their ability to care for mental health concerns in primary care. These insights informed the core objective for CAPP: to increase access to pediatric mental health care by building workforce capacity among pediatric PCPs.

In the Ideation phase in May 2019, CAPP facilitators organized 2 additional Stakeholder Workgroup meetings, during which we raised challenges identified during the Inspiration phase and then brainstormed possible solutions. Stakeholders provided feedback concerning: (1) which key PMHCA components to include at our program launch; (2) how to prepare PCPs to utilize program services; and (3) how to support PCPs in diagnosing and treating mental health concerns within primary care. After each brainstorming session, the facilitators met to organize the insights and to develop a prototype design for CAPP. Therefore, we designed CAPP’s core programs with pediatric workforce development as a central theme.

We launched the Implementation phase in September 2019 by conducting a 1-month pilot test of our prototype design at the pediatrics practice where we conducted the initial focus group. The prototype consisted of: (1) a warmline that provides PCPs with same-day telephone consultation with a child and adolescent psychiatrist, (2) an enrollment and orientation process to prepare PCPs to utilize CAPP services, and (3) training and education programs for PCPs. This pilot provided crucial early feedback about the consultation process and content. Additionally, we held an inaugural continuing medical education event on child and adolescent psychiatry topics for pediatric PCPs that allowed us to disseminate information on our services to community practitioners.

Given that the HCD process is iterative, we have cycled back through earlier phases to design new solutions as we expanded geographically. For example, we conducted a series of focus groups from April to August 2020 with PCPs from 11 practice sites contributing insights and feedback to further develop CAPP’s programmatic offerings (9). We discovered that clinician burnout prohibited engagement with CAPP services. As a result, we started to prioritize clinician well-being in our programming.

## 3 Results

### 3.1 Current programmatic elements and footprint

Today, like most other PMHCA’s across the US, CAPP’s core programmatic elements include: (1) PCP consultation with a child and adolescent psychiatrist via a telephone warmline; and (2) training and education, including continuing medical education (CME) for PCPs and school-based health providers through webinars and Project ECHO (Extension for Community Healthcare Outcomes) case discussions. Beyond these core services, we also started to provide limited direct services to patients and families, including Bridge Care Coordination to connect families to resources with the help of a licensed clinical social worker, as well as one-time Reach-Out-and-Connect (ROC) consultations with specialized UCSF psychologists for patients and caregivers regarding early childhood mental health, attention deficit hyperactivity disorder (ADHD) and behavior management, autism spectrum disorders, and eating disorders.

**Figure 2:**
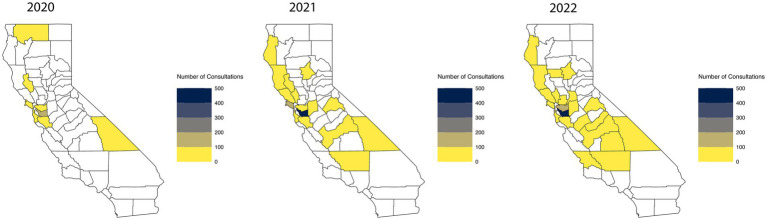
CAPP consultation calls by year and county.

Since its launch in September 2019, CAPP has grown geographically and numerically (Figure 2). As of August 2023, CAPP has enrolled 1,714 providers from 257 practices spread over 36 counties, and provided 3,288 consults for 586 unique providers on 2,703 unique lives. Clinical staffing includes a combined 1.7 full time equivalent (FTE) of child and adolescent psychiatrists, 0.25 FTE pediatrician, 0.65 FTE psychologist, and 1.0 FTE licensed clinical social worker. Research staff include a 0.05 FTE child and adolescent psychiatrist, 0.4 FTE health services researcher, and 1.0 FTE clinical research coordinator. Administrative staff include a full-time program manager, project analyst, and office associate.

### 3.2 Strategic partnerships and PCP engagement

Throughout the HCD process, multidisciplinary partnerships have been critical in providing end-user input to inform and improve program design. As an outgrowth of the initial HCD Stakeholder Workgroup, we created an Advisory Committee of general pediatricians, who provide ongoing end-user feedback. We also developed a larger, multidisciplinary Advisory Council, which provides programmatic and policy direction for CAPP’s activities. The Advisory Council consists of representatives from regional stakeholders like the California Children’s Hospital Association, California Primary Care Association (representing FQHCs statewide), the American Academy of Pediatrics (AAP)-California Chapter 1 (representing pediatricians in Northern California), California Children’s Trust (a state advocacy organization), the state of California Title V Maternal Child Health Division, and pediatric medical leaders from underserved communities across CAPP’s geographic catchment area.

This growing network of partnerships has proven essential for practice and provider adoption of CAPP. Much of CAPP’s outreach to PCPs has been conducted in collaboration with the AAP and other partners, who broadly advertised CAPP’s services to their members through publicity (e.g., newsletter posts, email blasts) and jointly organized outreach activities and events, including those offering continuing medical education (CME). Whether offered in collaboration with partners or solely through CAPP, CME credit has also proven to be an effective engagement tool for drawing in new PCPs and for increasing interaction with PCPs already enrolled in CAPP. This has allowed us to increase PCP awareness of CAPP’s services as well as confidence to identify and treat common pediatric mental health conditions.

### 3.3 Program evaluation results

Data on PCP consultations collected between September 2021 and September 2022 showed that the primary reasons for consultation requests were guidance on medications (65%) and selecting appropriate treatment (16%). Regarding patient diagnoses, PCPs most frequently consulted about patients with anxiety (29.4%), ADHD (24.3%), and depression (24.3%). Forty-eight percent of consults involved 2 or more psychiatric diagnoses.

Monthly feedback surveys were administered to PCPs who utilized CAPP services at least once in the preceding month. A total of 238 of 1,523 surveys (15.6%) were completed between July 2021 and April 2023 and demonstrated that the program was well-received by PCPs. PCPs responded to the 10 questions using a 5-point Likert scale (from 1 [strongly disagree] to 5 [strongly agree]). Average ratings for questions assessing appropriateness (e.g., “The CAPP consultant provides recommendations that are helpful to my patients”), feasibility (e.g., “I am able to consult a CAPP clinician in a timely manner”), and acceptability (e.g., “I have seen improvements in psychiatric symptomatology in my patients because of CAPP”) ranged from 4.43–4.75. Our assessment domains were derived from the Proctor framework (32).

## 4 Discussion

### 4.1 Lessons learned and limitations

This Community Case Study of the CAPP program offers key lessons regarding implementing and scaling a PMHCA program designed to close gaps in access to timely pediatric mental health care in California. In addition, this case study provides insights into using HCD processes to develop and implement a new PCMHA program within a large and socioeconomically diverse catchment area, despite widespread clinician burnout and systemic strain in the wake of the COVID-19 pandemic.

A key takeaway was the importance of using a people-centered approach and continuously cultivating trusting relationships. Throughout CAPP’s implementation, we learned that pediatric PCPs are more likely to consult with experts whom they know or trust. PCPs have shared that they “feel like an intern again” when first consulting with CAPP. Through timely, respectful, and culturally responsive interactions with individual PCPs, consultants develop an understanding of PCP needs, which is critical for building trust. Additionally, the consultant’s teaching and live coaching supports a PCP’s iterative increase in skill and confidence as they apply new knowledge with their patients. All consultation and education offerings must be accessible, practical, feasible—that is, within the scope of the PCP’s capability and confidence level and within the family’s practical and motivational capabilities.

This ethos of trust and community-building underlies CAPP’s consultative services, education, and outreach and advocacy efforts. Relationships built with PCPs during consultation and training have generated practice-level provider champions, who have proven to be linchpins for driving their peers’ engagement and utilization of CAPP. Community support is further established during CAPP’s Project ECHO, which adheres to Project ECHO’s hallmark “All Teach, All Learn” collaborative and interactive case-based learning structure.

In addition to partnerships with individual PCPs, multidisciplinary partnerships have proven critical in providing end-user input to inform and improve program design. Practice and system-level organizational leadership buy-in facilitated CAPP’s outreach and marketing and the development of workflows that enabled PCPs to utilize CAPP’s services. Relationships with leaders of healthcare systems, insurers, professional organizations, and advocacy groups have helped secure further funding and support for CAPP’s expansion. This growing network of partnerships, developed through the cultivation of personal relationships and trust over time, has proven essential for CAPP’s rapid growth and sustainability. It takes time to develop relationships and to expand networks across geographic and institutional barriers, and it takes time for practice habits and culture to change, especially in the face of potential barriers like stigma, PCP discomfort, billing expectations, and limited access to local mental health resources.

HCD emphasizes flexibility and adaptability throughout all phases, from design to implementation. Our pivot towards emphasizing clinician wellness in our day-to-day consultative and educational programs arose from the recognition of clinician burnout as a major factor affecting provider willingness to expand the scope of their clinical practice to include mental health. PCPs have shared feedback on effective components of consultation that supported their gains in knowledge and confidence. They have also provided input on preferred topics for educational sessions and offered specific workflow modifications, including the ability to self-schedule consults, integrate notes into electronic health records (EHR), and connect to more intensive specialty services. This last component is provided by CAPP’s licensed clinical social worker, who provides care coordination and resource navigation for under-resourced families. In all programmatic aspects, continuous end-user feedback, and adaptation have allowed for the flexibility to meet many of the needs of varied counties, healthcare systems, and practice types throughout California.

Despite such adaptability and flexibility in design and implementation, engagement and utilization has varied significantly by region, especially across FQHC networks, despite being designated as mental-health and medically underserved areas. Engagement seems to hinge most strongly on internal practice champions, followed by leadership support at the practice and system levels. One particular highly underserved area has been slower to engage, despite significant clinical need and strong individual practitioner participation and advocacy along with vigorous engagement efforts that included newsletters, direct telephone outreach to health system leaders, and in-person trainings. Regional variations in practice culture and billing expectations appear to be important factors, and for FQHCs in particular, lack of additional revenue for addressing complex and time-consuming concerns like mental health is a key barrier. While the cultivation of trust remains an important facilitator for program uptake, further progress in this area will also likely require state policy and regulatory change to provide greater financial incentives to address pediatric mental health.

CAPP has also run into limitations posed by ongoing challenges of incorporating time for consultation amid the busy clinical schedules typical of pediatric primary care. One strategy being considered by CAPP is asynchronous e-consults that allow secure email-like communication between PCPs and specialist consultants, particularly for more straightforward questions or follow-up questions to telephone consults.

Methodologically, this present study is not a formal evaluation of the CAPP program but rather offers insight into the approach that guided CAPP’s programmatic development; a mixed method evaluation is currently underway to assess program implementation processes and outcomes. Although this study incorporates a HCD approach for PMHCA program development, the utility and applicability of specific programmatic elements may not be generalizable to PMHCA programs in other geographic areas. Additionally, CAPP was developed and implemented in a community pediatric practice network affiliated with an academic medical center, which may not be representative of most practices. Thus, PMHCA development and implementation in other settings with different healthcare infrastructures and resources may require additional adaptations. Finally, while HCD proved helpful in encouraging stakeholder engagement and investment in program design, our sample of stakeholders was specifically chosen for their interest in building in a PMHCA program. Another limitation is the lack of access to clinical outcomes data needed to evaluate CAPP’s ability to achieve its ultimate goal of improving outcomes for youth facing a swelling mental health crisis.

Despite these important programmatic and methodological limitations, important lessons arise from CAPP’s experience in using HCD to adapt PMHCA program elements to Northern and Central California’s communities in a time of pressing need. Most importantly, this Community Case Study highlights the critical role of partnerships and the importance of taking a people-centered approach, as captured in CAPP’s motto, “Connecting for Care.”

## Data availability statement

The raw data supporting the conclusions of this article will be made available by the authors, without undue reservation.

## Ethics statement

The studies involving humans were approved by UCSF Human Research Protection Program. The studies were conducted in accordance with the local legislation and institutional requirements. The ethics committee/institutional review board waived the requirement of written informed consent for participation from the participants or the participants’ legal guardians/next of kin because an anonymous survey was given.

## Author contributions

CL: Writing – original draft, Conceptualization, Funding acquisition, Methodology, Supervision. JJ: Writing – original draft. JY: Writing – original draft. MF: Formal analysis, Visualization, Writing – original draft. PS: Supervision, Writing – original draft.
